# β-blocker Therapy is Not Associated with Reductions in Angina or Cardiovascular Events After Coronary Artery Bypass Graft Surgery: Insights from the IMAGINE Trial

**DOI:** 10.1007/s10557-015-6600-y

**Published:** 2015-06-14

**Authors:** Harmen G. Booij, Kevin Damman, J. Wayne Warnica, Jean L. Rouleau, Wiek H. van Gilst, B. Daan Westenbrink

**Affiliations:** Department of Cardiology, University Medical Center Groningen, Hanzeplein 1, P O Box 30001, 9700 RB Groningen, The Netherlands; Foothills Hospital, University of Calgary, 1403-29 Street NW, Calgary, AB T2N 2T9 Canada; Institut de Cardiologie de Montreal, University of Montreal, 5000 Belanger St East, Montreal, QC H1T1C8 Canada

**Keywords:** Coronary artery disease, Myocardial ischemia, Coronary artery bypass, Myocardial revascularization, Adrenergic beta-Antagonists

## Abstract

**Purpose:**

To evaluate whether β-blockers were associated with a reduction in cardiovascular events or angina after Coronary Artery Bypass Graft (CABG) surgery, in otherwise stable low-risk patients during a mid-term follow-up.

**Methods:**

We performed a post-hoc analysis of the IMAGINE (Ischemia Management with Accupril post–bypass Graft via Inhibition of angiotensin coNverting Enzyme) trial, which tested the effect of Quinapril in 2553 hemodynamically stable patients with left ventricular ejection fraction (LVEF) >40 %, after scheduled CABG. The association between β-blocker therapy and the incidence of cardiovascular events (death, cardiac arrest, myocardial infarction, revascularizations, angina requiring hospitalization, stroke or hospitalization for heart failure) or angina that was documented to be due to underlying ischemia was tested with Cox regression and propensity adjusted analyses.

**Results:**

In total, 1709 patients (76.5 %) were using a β-blocker. Patients had excellent control of risk factors; with mean systolic blood pressure being 121 ± 14 mmHg, mean LDL cholesterol of 2.8 mmol/l, 59 % of patients received statins and 92 % of patients received antiplatelet therapy. During a median follow-up of 33 months, β-blocker therapy was not associated with a reduction in cardiovascular events (hazard ratio 0.97; 95 % confidence interval 0.74–1.27), documented angina (hazard ratio 0.85; 95 % confidence interval 0.61–1.19) or any of the individual components of the combined endpoint. There were no relevant interactions for demographics, comorbidities or surgical characteristics. Propensity matched and time-dependent analyses revealed similar results.

**Conclusions:**

β-blocker therapy after CABG is not associated with reductions in angina or cardiovascular events in low-risk patients with preserved LVEF, and may not be systematically indicated in such patients.

## Introduction

Beta adrenoreceptor blockers (β-blockers) are among the most commonly prescribed cardiovascular drugs that are used to treat hypertension, arrhythmias, coronary artery disease (CAD) and heart failure. In patients with stable CAD, β-blockers are recommended as first line therapy based on their potent anti-anginal effects and on extrapolation of the prognostic benefits that has been demonstrated after myocardial infarction (MI) and in patients with heart failure [1, 2].

Most of the studies supporting the efficacy of β-blockers in patients with CAD predate the current era of coronary revascularisation, more intense anti-platelet therapy, the use of statins and more stringent blood pressure goals and were specifically designed to evaluate their effects on angina. While β-blockers are often continued in patients after coronary artery bypass grafting (CABG) surgery, even in patients with preserved left ventricular (LV) function, evidence for their efficacy in this setting is sparse [1, 3, 4]. This issue is particularly relevant since β-blockers may cause counterproductive side effects such as new onset diabetes and dyslipidemia [5, 6].

We therefore aimed to evaluate whether β-blocker therapy was associated with a reduced incidence of angina or cardiovascular events when continued after CABG. Therefore, we performed a post-hoc analysis of the IMAGINE (Ischaemia Management with Accupril post-bypass Graft via Inhibition of angiotensin coNverting Enzyme) trial database which comprised of low-risk patients with normal cardiac function, randomized to quinapril or placebo early after elective CABG surgery.

## Methods

A detailed description of the IMAGINE-trial protocol has been published previously [7]. In brief, it was a double-blinded, placebo-controlled, randomized, international, multicentre trial that tested whether the ACE-inhibitor quinapril when compared to placebo reduced symptoms of angina or cardiovascular events in patients with preserved LV function post-CABG surgery during a mid-term follow-up for a maximum of 43 months. Patients were included between November 1999 and September 2004. Written informed consent was obtained from all patients. The study was in compliance with the Declaration of Helsinki and the ethics committees from all participating institutions provided approval of the research protocol. In this trial, ACE-inhibition with quinapril did not improve outcome when started early after CABG in low-risk patients with preserved LV function, while adverse events were increased with quinapril during the first 3 months after randomization.

### Patients

In total, 2553 patients were randomized to quinapril or placebo within 7 days after scheduled CABG, except in France where randomization was possible until 10 days post-CABG. If tolerated, the ACE inhibitor quinapril was uptitrated to 40 mg daily or its placebo equivalent. Patients were eligible for participation when hemodynamically stable and if left ventricular ejection fraction (LVEF) was >40 %. Serum creatinine >2.26 mg/dL (200 μmol/L) was an exclusion criterium as were suspicion of renal artery stenosis, a single kidney or a transplanted kidney. During the study, type II diabetes with microalbinuria and insulin-dependent diabetes became exclusion criteria due to increasing evidence of benefit of ACE inhibitors in these patients.

### β-blocker Treatment

Of the 2553 patients included in the IMAGINE trial, 320 patients were using sotalol. Because sotalol has class 3 anti-arrhythmic effects which may modulate event rate through pro-arrythmic effects, patients using sotalol were excluded from this analysis. Thus, 2233 patients were available for analysis. Patients were divided in two groups, according to β-blocker therapy. β-blocker dose was expressed as a percentage of the maximum recommended dose. For the time-dependent analysis β-blocker use was scored at randomization, 50, 90 days, 1, 2 and 3 years after randomization.

### Endpoints

The primary IMAGINE endpoint consisted of the composite of cardiovascular death, resuscitated cardiac arrest, nonfatal MI, coronary revascularization, unstable angina requiring hospitalization, documented angina not requiring hospitalization, congestive heart failure which required hospitalization and stroke. The secondary IMAGINE endpoint consisted of the primary endpoint with the addition of transient ischemic attack and other cardiovascular events requiring hospitalization. One of the unique features of the IMAGINE trial was the meticulous verification of myocardial ischemia in patients with suspected recurrence of angina. An episode of angina was considered valid if typical symptoms of angina were associated with one of the following conditions: temporary ST deviations on electrocardiogram; a stress test with reversible wall motion abnormalities on echocardiography or reversible nuclear scan defects; coronary angiography demonstrating compatible lesions which could not be explained by incomplete revascularization or any episode of angina requiring hospitalization. Finally, we defined major adverse cardiovascular events (MACE) as the composite of angina, cardiovascular death, resuscitated cardiac arrest, nonfatal MI and coronary revascularization.

### Statistical Analysis

The baseline characteristics were compared according to presence of β-blocker therapy using students T-, Mann–Whitney U-, *χ*2- or Fisher exact tests, as appropriate. Time to first event was calculated by the Kaplan–Meier method and displayed graphically. Differences in event rate according to β-blocker therapy were calculated from a Cox proportional hazards regression model and expressed as an adjusted hazard ratio with two-sided confidence interval of 95 %. Cox regression analysis was adjusted for the effects of age, gender, ethnicity, history of MI, revascularization, non-cardiac vascular event, hypertension, diabetes, hypercholesterolemia, the number of days after surgery that the patient was randomized, beating heart surgery, nr of grafted vessels, complete revascularization, LVEF and concomitant medication. Propensity matched analysis was performed as a sensitivity analysis and as an additional effort to adjust for residual confounding. We calculated a propensity score for β-blocker-use with multivariable logistic regression, using all available variables. Covariates were selected when associated with β-blocker therapy or when they were independently associated with the outcome. Patients were then matched based upon β-blocker treatment and similar propensity score based on 1 to 1 nearest neighbor matching without replacement. Pre-match imbalance and post-match balance were estimated with standardized differences for each covariate. Since approximately 20-25 % of patients switched groups (started or discontinued a β-blocker) over time either permanent or temporary, we performed an additional Cox proportional hazards regression analysis with β-blocker as the time-dependent covariate. The software packages used for these analyses were SPSS 20.0 and STATA (version 12.0).

## Results

### Baseline Characteristics

Of the 2233 patients analyzed, 1709 (76.5 %) used a β-blocker. At 1 year, 1174 patients (62.0 %) used a β-blocker and 801 (58.9 %) 2 years after randomization. Average β-blocker dose was 41.2, 41.8 and 40.9 % of the maximal recommended dose at these time points respectively. The maximal recommended dose was given in 128, 103 and 67 patients respectively (7.5, 8.8 and 8.4 % respectively). Baseline characteristics are shown in Table 1. Overall, these were low-risk patients with mean age of 61 ± 10 years, mean LVEF 60 ± 10 %, low prevalence of diabetes (219 patients, 9.8 %), and good renal function (eGFR 69 ± 24 ml/min/1,73 m2). Patients treated with β-blockers had a history of hypertension more often (50 % vs 42 %) while other medical history was comparable between groups. Mean arterial pressure, heart rate and LVEF were also similar between groups. The majority of patients received statin therapy (53 and 60 % for no β-blocker and β-blocker patients respectively), while other lipid-lowering therapy was given in 3 % of patients. Patients on β-blocker therapy were more frequently receiving antiplatelet therapy and statins, but less frequently using anti-arrhythmic drugs or calcium channel blockers.

**Table 1:** Baseline characteristics stratified for use of β-blocker at randomization

Variable	no β-blocker (*n* = 524)	β-blocker (*n* = 1709)	*p*-value
General characteristics			
Age (yrs)	62 ± 10	61 ± 10	0.052
Male, n (%)	459 (88)	1495 (88)	1.000
Caucasian, n (%)	503 (96)	1645 (96)	0.169
Days post CABG	4.0 ± 1.7	4.3 ± 1.7	<0.001
Body mass index (kg/m^2^)	27.9 ± 5.0	27.6 ± 5.5	0.350
Medical history			
Myocardial infarction, n (%)	213 (41)	668 (39)	0.540
Coronary revascularization, n (%)	105 (20)	19.4 (19)	0.753
Non-cardiac vascular event, n (%)	60 (12)	182 (11)	0.630
Diabetes, n (%)	50 (10)	169 (10)	0.867
Hypertension, n (%)	222 (42)	854 (50)	0.002
Hypercholesterolemia, n (%)	411 (78)	1343 (79)	0.951
Current smoker, n (%)	100 (19)	349 (20)	0.260
Surgical characteristics			
Beating heart surgery, n (%)	90 (17)	318 (19)	0.478
Three vessel disease, n (%)	340 (65)	1090 (64)	0.845
Nr of anastomoses, n (%)	3.3 ± 1.1	3.2 ± 1.1	0.335
Complete revascularization, n (%)	470 (90)	1490 (87)	0.128
Hemodynamics			
Systolic blood pressure (mmHg)	122 ± 14	121 ± 13	0.042
Diastolic blood pressure (mmHg)	70 ± 9	70 ± 9	0.257
LVEF (%)	60 ± 10	60 ± 10	0.830
Heart rate (bpm)	83 ± 13	82 ± 13	0.207
eGFR (ml/min/1,73 m^2^)	68 ± 26	69 ± 23	0.258
Medication			
Quinapril, n (%)	263 (50)	856 (50)	1.000
Anti-arrhytmic drug, n (%)	87 (17)	134 (8)	<0.001
Calcium-channel blocker, n (%)	198 (38)	372 (22)	<0.001
Cardiac glycoside, n (%)	29 (6)	97 (6)	1.000
Diuretic, n (%)	175 (33)	589 (35)	0.674
Coumarine derivate, n (%)	49 (9)	70 (4)	<0.001
Antiplatelet, n (%)	453 (87)	1610 (94)	<0.001
Other lipid-lowering drugs, n (%)	11 (2)	46 (3)	0.528
Statin, n (%)	280 (53)	1032 (60)	0.005
Nitrate, n (%)	39 (7)	100 (6)	0.752

Values shown are means ± SD or n (%). *CABG* Coronary Artery Bypass Grafting, *eGFR* Estimated Glomerular Filtration Rate, *LVEF* Left Ventricular Ejection Fraction

Mean LDL values were slightly higher at randomization in patients without β-blocker (3.0 ± 1.0 vs 2.8 ± 1.0, *p* = 0.008) but did not differ during follow-up (2.6 ± 0.8 vs 2.6 ± 0.8 mmol/l, *p* = 0.50 at 1 year follow-up and 2.5 ± 0.8 vs 2.5 ± 0.8 mmol/l, *p* = 0.72 at study closure). Mean systolic blood pressure was 121 ± 14 mmHg and slightly lower in patients on β-blockers at randomization, but it remained ≤131 mmHg with no significant difference between groups throughout follow-up. In the majority of patients (88 %) revascularization was complete (defined as bypass of all stenosis of >70 % in vessels with a diameter >1 mm). Surgical characteristics were comparable.

### Cox Regression Analysis

**Fig. 1:**
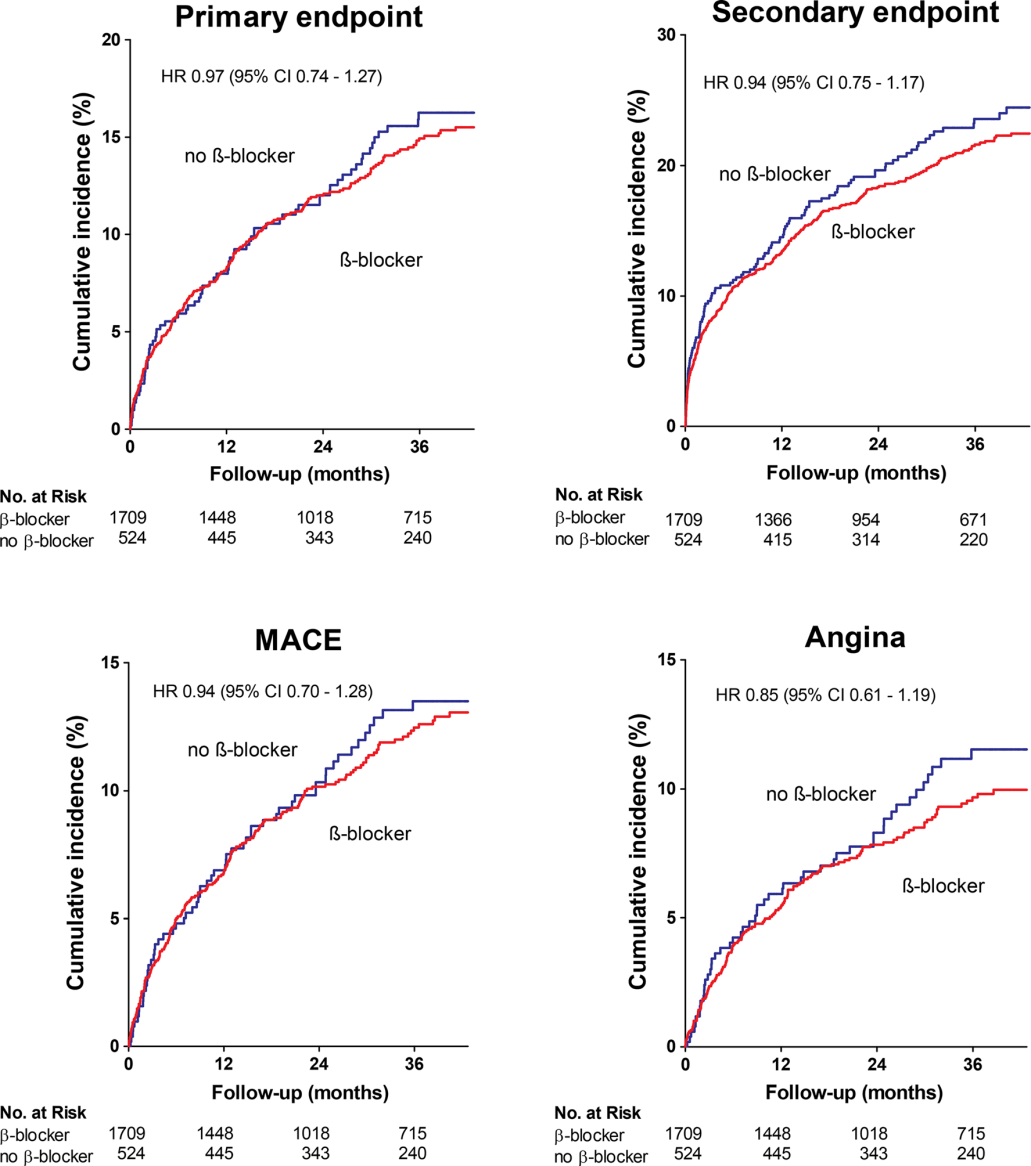
Outcome according to β-blocker therapy – Cumulative event rates for composite endpoints stratified for β-blocker therapy. Hazard ratios are adjusted for age, gender, ethnicity, history of myocardial infarction, revascularization, non-cardiac vascular event, hypertension, diabetes, hypercholesterolemia, days after CABG (coronary artery bypass grafting), beating heart surgery, nr of vessel disease, complete revascularization, left ventricular ejection fraction and concomitant medication. MACE, Major Adverse Cardiovascular Event

**Fig. 2:**
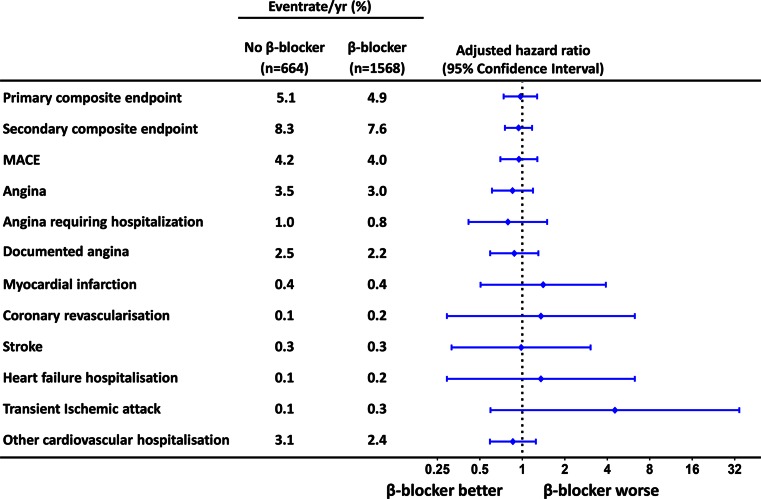
Cox regression – Hazard ratios and 95 % confidence intervals for composite endpoints and individual components after adjustment for same variables as in Fig. 1. MACE, Major Adverse Cardiovascular Event

**Fig. 3:**
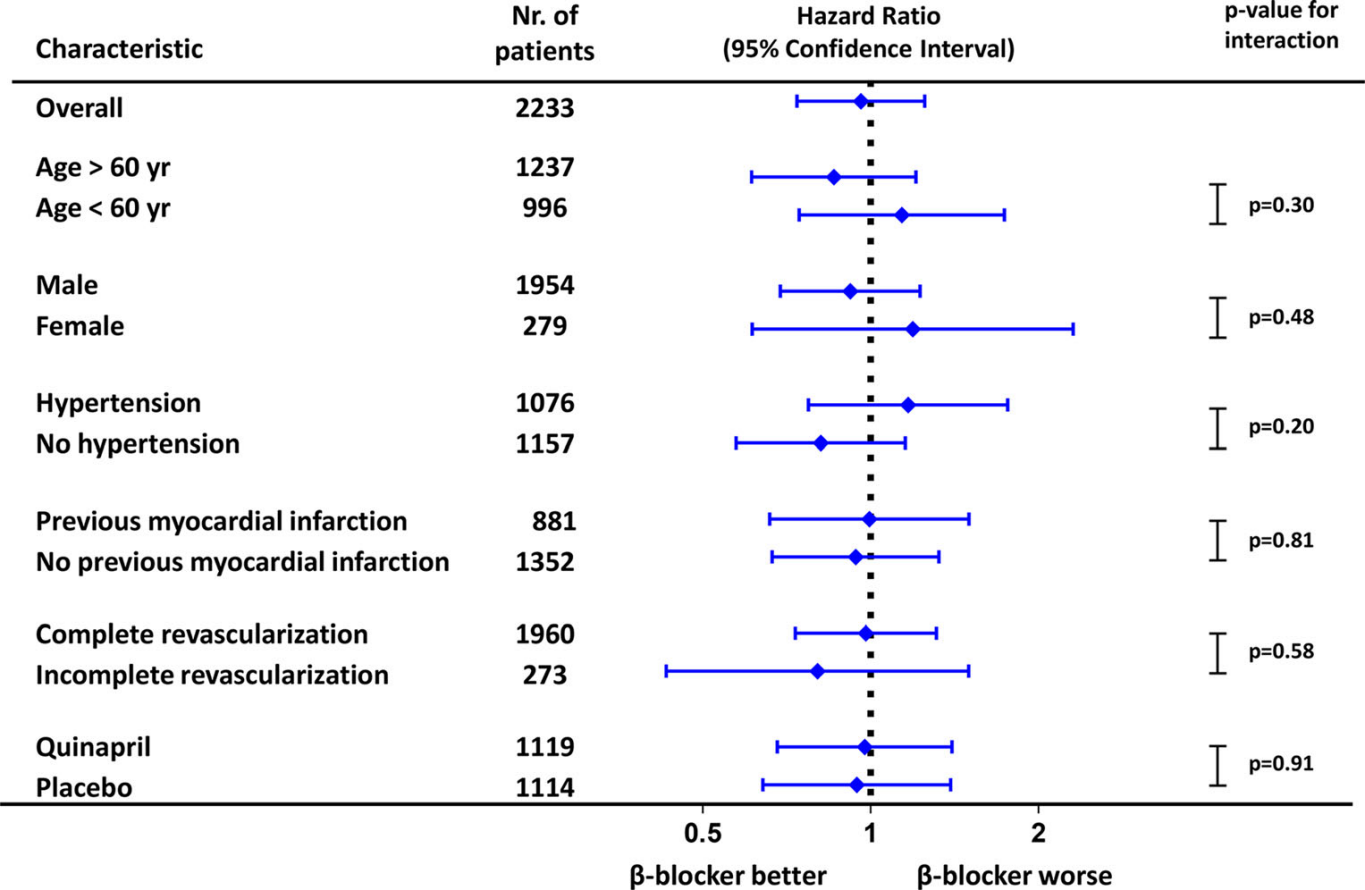
Interaction analysis for β-blocker – Hazard ratios for β-blocker therapy in relevant subgroups

Out of the 2233 patients analysed, 299 (13.4 %) patients had experienced a primary event, while 451 (20.2 %) patients had experienced a secondary event during a median follow-up of 33 months (IQR 16–43). Total event count for MACE and Angina was 245 (11.0 %) and 191 (8.6 %) respectively. β-blocker treatment was not associated with a difference in cumulative incidence of any of the composite endpoints (primary endpoint, secondary endpoint, MACE, angina, (Fig. 1)). Multivariate regression did not reveal any association between β-blocker treatment and the primary endpoint (hazard ratio (HR) 0.97; 95 % confidence interval (CI) 0.74–1.27), documented angina (HR 0.85; 95%CI 0.61–1.19) or any of the other composite endpoints and their individual components (Fig. 2). The neutral effects of β-blocker therapy were consistent among several relevant subgroups including age, gender, hypertension, previous MI, completeness of revascularization and treatment allocation (Fig. 3).

### Propensity Matched and Time-Dependent Analysis

**Fig. 4:**
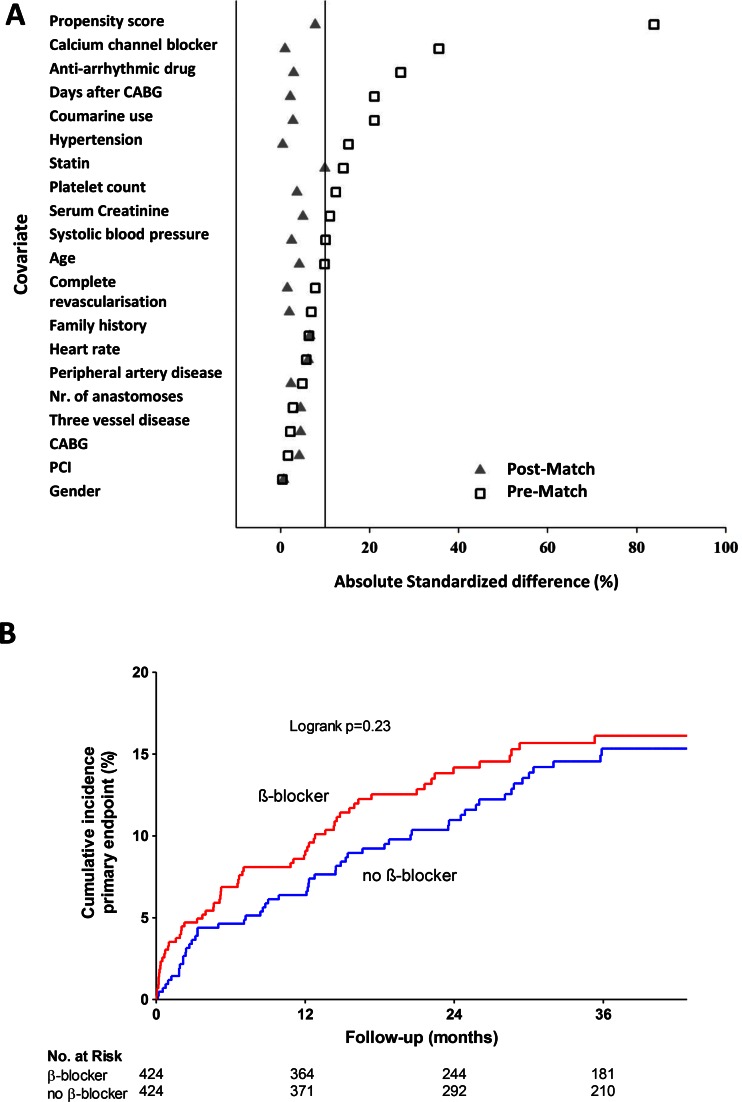
Propensity matched analysis – (**a**) standardized differences between baseline characteristics before and after matching. (**b**) Cumulative event rate for the primary endpoint in the propensity matched population. CABG, Coronary Artery Bypass Grafting; PCI, Percutaneous Coronary Intervention

**Fig. 5:**
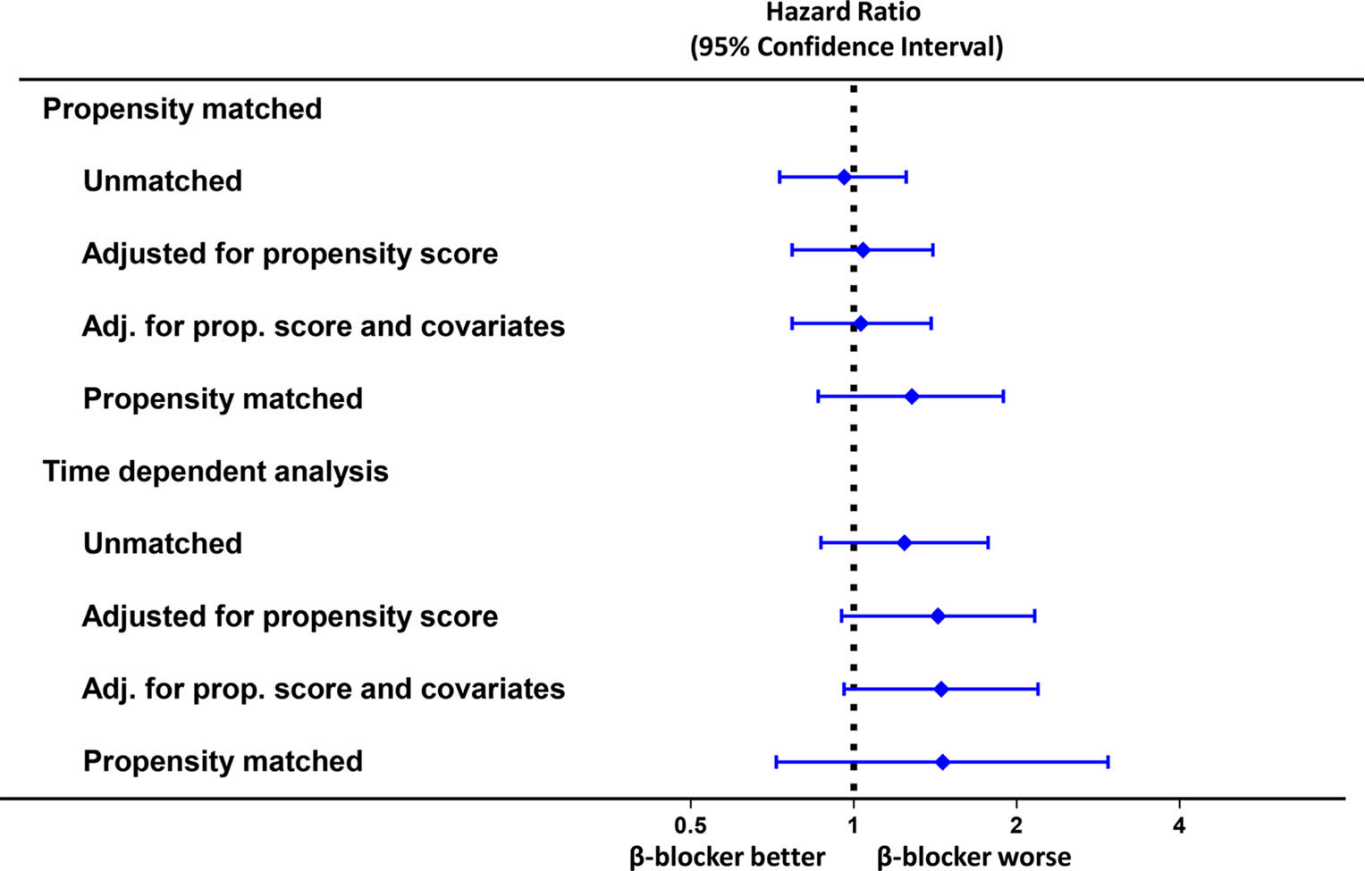
Risk for primary endpoint with propensity score and time-dependent analysis of β-blocker therapy - Analysis performed with β-blocker therapy at randomization and β-blocker as a time-dependent covariate

The propensity matched population consisted of 424 patients per group. Absolute standardized differences for all baseline-characteristics were <10 %, indicating an adequate match (Fig 4a). There was no association between β-blocker therapy and the occurrence of the primary IMAGINE endpoint when adjusting for propensity score and its covariates in the unmatched population nor when the propensity matched population was considered separately (Figs. 4b and 5). Similar results were obtained for the secondary endpoint, MACE and angina (data not shown). To account for differences in treatment over time, we analysed β-blocker therapy as a time-dependent covariate in our Cox-regression models. Again, no association was found for β-blocker therapy and outcome (Fig. 5).

## Discussion

β-blocker therapy has been the cornerstone of pharmacotherapy of CAD for decades, but recent evidence suggests that this central role may not be justified in patients that are at relatively low risk, have good control of their cardiovascular risk factors and are receiving evidence-based therapy [5, 8, 9]. In patients receiving CABG, pre-operative β-blocker therapy has been reported to be as high as 80–93 % over the last few years [9]. However, it is unknown if β-blockers should be continued after CABG. We therefore performed an explorative analysis to determine if β-blocker therapy was associated with reductions in the incidence of angina or cardiovascular events after CABG surgery in stable patients without heart failure or LV dysfunction. Additionally, we investigated the incidence of individual components of the composite outcome.

In our current retrospective analysis of low-risk patients, we show that β-blockers were frequently continued after CABG for a mid-term follow-up to 43 months. β-blocker use was, however, not associated with a decreased risk of recurrent angina or cardiovascular events, nor any of the individual components of the composite outcomes. Our results were consistent across different types of analyses, including propensity matched and time-dependent analyses, suggesting that the lack of association between β-blocker therapy and clinical outcome is robust. This patient population was initially selected to test whether ACE-inhibition in patients with a low event-rate would have additional benefit on top of standard medical therapy. This low event-rate did naturally reduce the power of our analysis, but also indicates the potential benefit of β-blockers in this population is limited. While the results may be different in the general CABG population, our current data fuel the hypothesis that these agents should not be continued indiscriminately.

### β-blockers in Patients with CAD

β-blockers reduce heart rate, blood pressure and stroke volume, three key determinants of myocardial oxygen demand. In addition, β-blockers increase coronary blood flow through prolongation of the diastole. In concert, these mechanisms are deemed responsible for the beneficial effects of β-blockers. Indeed, several studies have convincingly demonstrated that β-blockers reduce the burden of angina in patients with obstructive CAD [2, 10]. The prescription of β-blockers in patients with CAD is, however, more generally advocated based on the extrapolation of prognostic benefit observed in patients after MI and in heart failure patients [2]. Our study suggests that in low-risk patients after CABG surgery, these extrapolations may need additional investigation.

### β-blocker Therapy After Revascularization

Several potential explanations may underlie the neutral effects of β-blocker therapy in our population. First, most patients were fully revascularized, thereby effectively removing the substrate for angina and potentially for cardiovascular events as well. Therefore the potential benefit of β-blockers seems less relevant for the occurrence of these events. Our findings are in line with an earlier study which showed no benefit of metoprolol on exercise capacity or myocardial ischemia in patients revascularized with CABG [11].

Second, this study purposely selected CAD patients with low risk for cardiovascular events. Indeed, cardiovascular mortality was <1.4 % over the median follow-up of 3 years, and the incidence of MACE was only 9.4 %. This is similar to patient populations with cardiovascular risk factors, but without established CAD, underscoring the low incidence of cardiovascular events in the present population [5, 12]. In fact, the recently published Study assessInG the morbidity-mortality beNefits of the If inhibitor ivabradine in patients with coronarY artery disease (SIGNIFY) which randomized 19 102 patients with normal cardiac function to the selective sinus node inhibitor ivabradine, failed to show a benefit on outcome [13]. Heart rate reduction is considered to be the most important mode of action of β-blockers in CAD. The neutral results of SIGNIFY therefore provide an additional line of evidence supporting the concept that modulation of the sympathetic tone is not generally effective after revascularization in low-risk patients. Of note, patients using β-blockers have significantly lower heart rates compared to the reference group. β-blockers are particularly effective in patients with LV dysfunction [14], which was previously common after MI. A recent study reported a mean LVEF of 54.8 % in STEMI-patients who had received primary percutaneous coronary intervention [15]. In an analysis of contemporary patients with history of MI, β-blockers were not associated with a reduction in cardiovascular events [5]. Other studies only found a favorable association between β-blockers and cardiovascular events in patients with recent MI [16–18]. Together these findings suggest that the protective effects of β-blockers are confined to patients with a recent MI, ongoing myocardial ischemia or significant LV dysfunction.

### Clinical Implications

The results of this analysis of a low-risk population with normal cardiac function suggest that β-blockers do not have additional beneficial effects after CABG. This has not been studied in a prospective randomized trial. Therefore, there are no data supporting indiscriminate use of β-blockers in patients who are asymptomatic, are receiving evidence-based therapy for CAD and with good LV function after successful revascularization. This is reflected in the most recent AHA guidelines for management of stable ischemic heart disease with a class IIb recommendation for these patients [19]. The ESC guidelines do not mention a specific recommendation for β-blocker use in these low-risk asymptomatic patients [1]. Although β-blockers are still important drugs for the treatment of angina, recent MI and patients with LV dysfunction [10, 14, 16–18, 20–22], their efficacy in other indications is under scrutiny. Controversy has risen about the effectiveness of β-blockers during non-cardiac surgery [23], although it is still strongly recommended (class I) to continue β-blockers in patients who are already receiving these drugs [24].

### Limitations

The current analysis is essentially a retrospective analysis of prospectively collected data and, despite multivariate adjustments and propensity matching, residual confounding can never be fully eliminated. One might argue that the lack of benefit was partly caused through bias by indication. Patients on β-blockers might have been patients with a higher cardiovascular risk as these agents are often prescribed for residual angina, hypertension, atrial fibrillation or MI. On the contrary, patients not using β-blockers were treated with other anti-anginal drugs and had a slightly higher cardiovascular risk with higher LDL-cholesterol levels and a little less often anti-platelet therapy. Despite this apparently higher cardiovascular risk in the reference group, β-blockers were not associated with benefit in our analysis. Moreover, our analysis was adjusted for cardiovascular risk as rigorously as possible, including propensity matching. Another potential limitation of our analysis is that the sample size might be too low. Although this may true, we did not observe a trend towards an effect. In addition, the adequately powered SIGNIFY trial, which tested a drug with a similar mechanism, did not demonstrate any beneficial effect despite a large sample size of 19 102 patients [13]. Considering the very low event rate in our analysis, a trial twice the size of SIGNIFY would be required to answer this question. Even if β-blockers would appear effective, the high number needed to treat would most likely not outweigh the risks and side effects.

Our results should be regarded as hypothesis generating. Consequently, firm treatment recommendations based only on the current analysis should be avoided. In addition, we investigated a low-risk population, with good control of their risk factors, and receiving evidence-based therapy for CAD. The results for a similar analysis in the general CABG population may be different. Nevertheless, as evidence to support the continuation of β-blockers after revascularization is currently absent, our analysis generates the hypothesis that general application of β-blockers to patients after CABG might not be justified.

## Conclusions

β-blocker treatment after CABG in low-risk patients with preserved LV systolic function, good control of risk factors, and receiving evidence-based therapy, was not associated with a reduced incidence of cardiovascular events or angina during a median follow-up of 32 months.
